# Subclinical carotid vascular disease and risk factors for atherosclerosis in type 1 and type 2 diabetes

**DOI:** 10.1590/2359-3997000000264

**Published:** 2017-04-01

**Authors:** Viviane Z. Rocha, Raul D. Santos

**Affiliations:** 1 Instituto do Coração Faculdade de Medicina Universidade de São Paulo São Paulo SP Brasil Instituto do Coração (InCor), Faculdade de Medicina da Universidade de São Paulo, (FMUSP), São Paulo, SP, Brasil; 2 Centro de Medicina Preventiva Hospital Israelita Albert Einstein São Paulo SP Brasil Centro de Medicina Preventiva e Programa de Cardiologia, Hospital Israelita Albert Einstein (HIAE), São Paulo, SP, Brasil

The association of diabetes mellitus, both type 1 (T1DM) and type 2 (T2DM), with increased cardiovascular risk is well known (1), but growing evidence has shown a wide heterogeneity in the risk of cardiovascular events in diabetics. Besides clinical evaluation, assessment of subclinical vascular disease, such as screening for coronary artery calcium (CAC), a surrogate marker of atherosclerosis in those with normal renal function, and carotid intimal-media thickness (CIMT), can further stratify risk in diabetics, identifying higher and lower risk groups (2). In a sub-study from MESA (Multi-Ethnic Study of Atherosclerosis), for instance, the cardiovascular event rates in individuals with metabolic syndrome or T2DM were as low as in those without these conditions, unless CAC or CIMT were significantly elevated.

In this issue of *Archives of Endocrinology and Metabolism*, two studies unravel the predictors and risk associated with elevation of CIMT and presence of carotid plaque in two distinct groups of diabetic patients. While a universal concept of “cardiovascular risk equivalent” for the entire diabetic population has been progressively replaced for a more selective categorization within this group, understanding the utility of subclinical disease markers in this context, particularly subclinical carotid disease, still lacks further evidence.

In the first study in this issue, Kupfer and cols. (3) assessed the clinical predictors of subclinical carotid vascular disease in asymptomatic young adult women with T1DM. Of importance, cardiovascular events are more common and occur earlier in patients with T1DM compared to non-diabetics, particularly in women. In the General Practice Research Database (GPRD) from the UK, with over 7,400 T1DM patients and mean DM duration of 15 ± 12 years, T1DM male patients presented an absolute cardiovascular risk similar to that of men from general population 10-15 years older; type 1 diabetic women had an even greater risk compared to their non-diabetic counterparts (4). Therefore, young female type 1 diabetics, evaluated in the study by Kupfer and cols. (3), represent a subgroup that might benefit more importantly from preventive strategies, especially if they already present at a young age any sign of incipient atherosclerosis. In this study, 45 asymptomatic type 1 diabetic women, still young (mean age 36.2 ± 9.5 years), but with already a long-term DM duration (mean 18.1 ± 9.5 years), exhibited a mean CIMT of 0.25 mm, with presence of carotid plaques in 13%. CIMT, as measured by carotid artery ultrasonography, is the distance from the lumen-intima interface to the media-adventitia interface of the artery wall. The presence of carotid plaque is defined usually as an intima-media thickness of more than 1.5 mm. Unless there is a media layer hypertrophy due to uncontrolled hypertension, CIMT is usually recognized as a surrogate measure of atherosclerosis, and an independent predictor of cardiovascular outcomes (5). Moreover, the maximum IMT of the internal carotid artery and the presence of plaque in the same site improved significantly, yet modestly, the predictive power of Framingham risk factors for cardiovascular events (5). In T1DM, several risk factors, such as age, total cholesterol, LDL-C, smoking, as well as diabetes-related risk factors, including body mass index (BMI), DM duration, and albuminuria, also associate with CIMT and presence of plaque (6). The current study reaffirms the importance of some of these risk factors in subclinical carotid disease particularly in women. Hypertension and reduced insulin sensitivity, measured by the estimated glucose disposal rate (eGDR), were both correlated with CIMT and presence of plaque. T1DM women with carotid plaque were also older and with a trend toward higher albuminuria relative to the ones without plaques. The relative small sample size, cross-sectional study nature and the absence of a multivariate analysis are some of the limitations of this study and might help explaining the lack of association of CIMT/plaque with other important cardiovascular risk factors in diabetes. Interestingly, mean CIMT (0.25 ± 0.28 mm) in this study was low compared to the same measurement in other T1DM cohorts, even younger ones (7). However, as previously discussed, the comparison of CIMT values across different cohorts is highly challenging, considering distinct sample characteristics, and use of different techniques for measurement (8).

In the second study in this issue by Masson and cols. (9), the authors evaluated in a cohort of Argentinian type 2 diabetics the association of carotid plaques with cardiovascular disease risk estimated by distinct risk engines. The prevalence of carotid plaque in the whole cohort was high (51%), and was significantly greater in the top quartiles of calculated risk in all assessed scores. The higher occurrence of carotid plaques in this study compared to the one by Kupfer and cols. is not surprising, considering the more advanced age, the male majority, and the overall worse risk profile presented by the type 2 diabetics in the study by Masson and cols. The power of all tested scores to discriminate between those diabetics with and those without carotid plaques was reasonably good and was performed by robust C statistic comparisons (10). Ranging from a value of 0.5 (no discrimination) to 1.0 (perfect discrimination), the C statistic represents the probability that the predicted risk is higher for a case (“case” in the present study means “to have a carotid plaque”) than for a non-case (“non-case” in the present study means “not to have a carotid plaque”). In the work by Masson and cols., all areas under the ROC (receiver operating characteristic) curves were above 0.75, values that are considered as good discriminators. Since the discrimination with the use of scores is never perfect (11), it is expected a certain degree of disagreement between the calculated risk and presence of carotid plaques. Indeed, the prevalence of carotid plaque in the lowest quartile of all tested scores ranged from 9 to 19%, suggesting that a low score cannot rule out the possibility of a carotid plaque in these patients. Therefore, it is reasonable to think that the use of subclinical cardiovascular disease imaging could be especially valid in these estimated lower-risk diabetics to help identifying the more susceptible individuals despite the relative lack of a great risk factor burden (11). Overall, the calculation of the optimal cutoff point (OCP) for each score yielded values close to the actual thresholds used to classify patients as high risk (around 20% in 10 years). Nevertheless, the OCP for occurrence of carotid plaque of the score proposed by the 2013 ACC/AHA guideline was 14.3%, much higher than the threshold of 7.5% in 10 years used to stratify diabetics in the highest-risk category. Whether this discrepancy would result in overtreatment of those individuals with lipid lowering drugs, remains to be determined, since the presence of carotid plaque is only a surrogate marker of atherosclerosis and does not necessarily mean that a given patient will present a cardiovascular event in the long-term.

Several studies have demonstrated, in the general population and in diabetic patients, the superiority of CAC over CIMT as a predictor of coronary heart disease (CHD) and cardiovascular disease (CVD) events. An analysis from the MESA cohort also found that CAC improved prediction of CHD and CVD more than high CIMT or presence of carotid plaque (12). For stroke or transient ischemic attack, CAC and carotid parameters performed similarly (12). In individuals with metabolic syndrome or diabetes, CAC was also a better predictor of CHD and CVD events as compared to CIMT (2).

Despite the consolidation of cardiovascular risk heterogeneity among diabetics, stratified either by a risk score or imaging methods, further investigation should define whether a differential therapeutic approach based on calculated risks or high CIMT/CAC will translate in clinical outcome benefit.
